# Sinonasal Complications Following the Sinus Lift Procedure

**DOI:** 10.31486/toj.22.0125

**Published:** 2023

**Authors:** Jakob L. Fischer, Charles A. Riley, Ashutosh Kacker

**Affiliations:** ^1^ Department of Otolaryngology, Walter Reed National Military Medical Center, Bethesda, MD; ^2^ Department of Surgery, Uniformed Services University of the Health Sciences, Bethesda, MD; ^3^ Department of Otorhinolaryngology, Weill Cornell Medical College, New York, NY

**Keywords:** *Maxillary sinusitis*, *sinus floor augmentation*, *sinusitis*

## Abstract

**Background:** Although the incidence of postoperative acute and chronic rhinosinusitis in patients undergoing a sinus lift procedure is relatively high, a paucity of rhinology literature examines the management of and outcomes for this patient population. The objective of this study was to review the management and postoperative care of sinonasal complications and identify possible risk factors that should be considered prior to and following sinus augmentation.

**Methods:** We identified sequential patients who had undergone a sinus lift procedure and were referred to the senior author (AK) at a tertiary rhinology practice for intractable sinonasal complications and reviewed their charts for demographic data, history of illness including prereferral treatment, examination findings, imaging results, treatment modalities, and culture results.

**Results:** Nine patients were initially treated medically without improvement and subsequently underwent endoscopic sinus surgery. The sinus lift graft material remained intact in 7 patients. Two patients had extrusion of the graft material into the facial soft tissues, resulting in facial cellulitis requiring graft removal and debridement. Seven of the 9 patients had predisposing factors that could have prompted referral to an otolaryngologist for optimization prior to sinus lifting. The mean follow-up was 10 months, and all patients had full resolution of symptoms.

**Conclusion:** Acute and chronic rhinosinusitis is a complication of the sinus lift procedure and is more commonly seen in patients with preexisting sinus disease, anatomic sinonasal obstruction, and Schneiderian membrane perforation. Preoperative evaluation by an otolaryngologist may improve outcomes in patients at risk of sinonasal complications from sinus lift surgery.

## INTRODUCTION

Implant-supported prostheses have become a common means of replacing missing teeth and have reliable long-term results,^1^ but implant placement can be uniquely challenging along the posterior maxillary dentition. Because of bone atrophy and pneumatization of the maxillary sinus toward the alveolar crest, inadequate bone height can complicate osseointegrated implant placement.^2^ To address these issues, Tatum first described the sinus lift procedure in 1976 as a means to elevate the floor of the maxillary sinus, add bone height, and allow dental implantation.^3^ The procedure and its variations have been well-documented, ranging from a lateral approach via a maxillary sinus osteotomy to an internal approach using bone compression kits, with placement of autogenous, allogenic, or alloplastic material in a submucoperiosteal pocket.^1^

While the sinus lift procedure has been shown to be safe and effective, numerous complications related to the procedure have been reported, including perforation of the sinus membrane, oroantral fistula, poor osseointegration, graft migration or extrusion, graft infection, maxillary sinus mucoceles, and acute and chronic rhinosinusitis.^4^ Perforation of the Schneiderian membrane, the mucosal lining of the maxillary sinus, is reported to be the most common complication of the sinus lift procedure. The majority of early studies examining the role of Schneiderian membrane perforations concluded that the perforations likely did not result in increased secondary complication rates.^5-8^ The exception is a 2014 study by Nolan et al who noted Schneiderian membrane perforation was associated with a decrease in implant survival in their patient population.^9^ More recent studies have found associations between Schneiderian membrane perforation and higher rates of postoperative sinusitis and secondary infection.^4,10,11^

Acute maxillary sinusitis has been reported in 10% to 20%^6,9,12^ of patients after the sinus lift procedure, while 4% to 8%^4^ of patients reported symptoms consistent with chronic rhinosinusitis. The greatest risk factors associated with development of sinusitis appear to be Schneiderian membrane perforation,^8,9^ perioperative tobacco use, and former history of sinus disease.^13^ Timmenga et al noted that maxillary sinus physiology is affected by relocation of the antral floor in combination with mucosal trauma, inflammation, and/or fluid collection that may impair natural drainage.^14^ Still, preoperative otolaryngologic evaluation and computed tomography (CT) imaging of the sinuses are not routinely performed for sinus lift candidates unless they present with risk factors for postoperative sinusitis.^12-14^ The rhinology-specific literature examining this patient population and their outcomes is limited.

We assessed 9 patients with significant sinonasal complications after sinus lift surgery. The aim of the study was to review the management and postoperative care of sinonasal complications, examine the relationship between sinus lift graft migration and sinus complications, and identify risk factors that might predict otolaryngologic evaluation and intervention both before and after sinus lift surgery.

## METHODS

The New York Presbyterian Hospital-Weill Cornell Medical College Institutional Review Board approved the research protocol. Consecutive adult patients who underwent sinus lift surgery between 2005 and 2009 and who were referred to the senior author (AK) were included in this study. All patients included in the study cohort were referred to the senior author with a combination of symptoms including ipsilateral maxillary sinus pain/pressure, purulent rhinorrhea, and/or postnasal drip. All patients had at least partial opacification of the ipsilateral maxillary sinus on CT either as part of their prereferral workup or after initial clinical evaluation. We reviewed the electronic medical records of these patients and extracted demographic data, history of present illness including prereferral treatment, examination findings, imaging results, treatments, culture results, and the results of histopathologic evaluation. All data were entered into a deidentified database prior to analysis. Descriptive statistics were followed by univariate analysis of group differences among categorical variables as appropriate.

## RESULTS

A total of 9 patients were included in the study cohort, 6 female and 3 male patients, with an average age of 60 years (range 32 to 78 years). All patients were medically treated by their referring oral surgeons prior to referral. Prereferral treatment included oral antibiotics for all patients, with nasal steroids, antihistamines, and decongestants only used intermittently. Bone grafting material and sinus lift surgery evaluation records were not available for review because of limitations in access to dental and procedural records.

Initial rhinology evaluation included nasal endoscopy and CT scans of the paranasal sinuses (Table 1). Patient evaluation was notable for a deviated nasal septum in the majority of patients (7/9, 77.8%). One patient had a concha bullosa, a pneumatized middle turbinate. No other anatomic abnormalities were noted that would have potentially contributed to impaired sinus drainage and mucociliary clearance. All patients had unilateral maxillary mucosal thickening with at least partial opacification on the side of the sinus lift procedure. No patient had any evidence of direct obstruction of the ostiomeatal complex by graft material. Oroantral fistulas were identified in 4 (44.4%) patients. Two patients (22.2%) had migration of the bone graft to the soft tissues of the cheek that resulted in extensive facial cellulitis in both patients (Figure).

**Table 1.** Rhinology Evaluation Results After Referral for Complications of Sinus Lift Surgery, n=9

Finding	n (%)
Deviated nasal septum	7 (77.8)
Concha bullosa	1 (11.1)
Unilateral maxillary mucosal thickening and opacification	9 (100)
Oroantral fistula	4 (44.4)
Migration of bone graft	2 (22.2)

**Figure.**
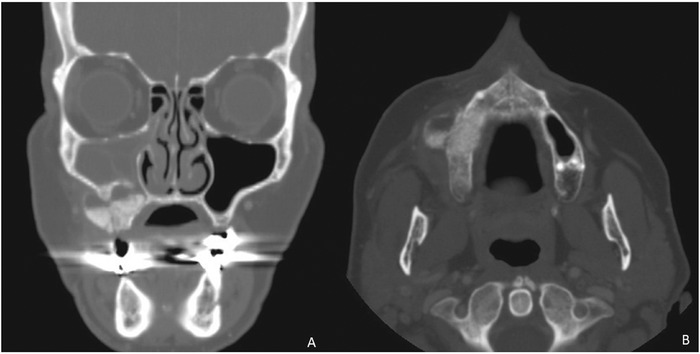
(A) Coronal and (B) axial computed tomography scan of a patient who had migration of the sinus lift graft material into the soft tissues of the cheek, resulting in extensive facial cellulitis and requiring debridement.

All patients required surgical intervention for the management of sinonasal disease. Endoscopic sinus surgery was performed in all patients, and 3 (33.3%) required septoplasty for a deviated nasal septum that was considered obstructive of the ostiomeatal complex. A septoplasty was performed if the degree of septal deviation would impair postoperative debridement or if the deviation was severe enough to impair drainage through wide maxillary antrostomies. Of the 2 patients who developed facial cellulitis secondary to graft migration into the soft tissues of the face, 1 patient was a posttransplant patient on immunosuppression. Both patients required surgical debridement and removal of the bone graft via an open approach, a Caldwell-Luc incision.

Cultures were taken from the involved maxillary sinus in all 9 patients, and 7 were polymicrobial (Table 2). The most commonly cultured pathogens were *Viridans streptococci* (44.4%) and *Staphylococcus aureus* (33.3%). Two of the 9 patients demonstrated no growth of bacteria from their maxillary sinus cultures.

**Table 2.** Maxillary Sinus Culture Results, n=9

Pathogen	n (%)
*Viridans streptococci*	4 (44.4)
*Staphylococcus aureus*	3 (33.3)
*Actinomyces* spp	1 (11.1)
*Peptostreptococcus* spp	1 (11.1)
*Stomatococcus* spp	1 (11.1)
*Capnocytophaga* spp	1 (11.1)
*Enterobacter* spp	1 (11.1)
*Propionibacterium acnes*	1 (11.1)

Note: Seven patients were polymicrobial, and 2 patients demonstrated no growth of organisms.

Histopathologic examination of tissue obtained during endoscopic sinus surgery demonstrated acute and chronic inflammation consistent with sinusitis in all 9 patients. Two of the 9 specimens demonstrated evidence of osteomyelitis, while none of the specimens demonstrated evidence of foreign body reaction.

Postoperative follow-up ranged from 1 week to 44 months, with a mean follow-up period of 10 months. All patients received postoperative culture-directed antibiotics and underwent postsurgical debridement at their 1-week follow-up appointments. At that first postoperative visit, 8 (88.9%) patients noted complete resolution of sinus symptoms. The remaining patient noted persistent facial pain that resolved with additional oral antibiotic treatment by 2 weeks after the initial surgery.

## DISCUSSION

The sinus lift procedure has undergone many revisions in technique and practice since its introduction in the 1970s by Tatum^3^ and remains an important means of dental rehabilitation.^1^ Despite changes and improvement in techniques over time, complications relating to the procedure remain relatively common and may have implications for oral surgeons and otolaryngologists.

The most common complication relating to the sinus lift procedure is perforation of the Schneiderian membrane along the floor of the maxillary sinus, generally estimated to occur in 20% to 25% of patients, although some studies report perforation rates as high as 42%.^4,9,15^ Perforation of the Schneiderian membrane has significant relevance for both the oral surgeon performing the sinus lift and the otolaryngologist who may see the patient postoperatively.

For the oral surgeon, patients with Schneiderian membrane perforation have been reported to have an increased rate of graft failure compared to patients with an intact membrane at the conclusion of surgery: 11.3% vs 3.4%, respectively.^9^ However, a 2016 meta-analysis examining complications related to Schneiderian membrane perforation found no significant difference in implant survival between perforated and nonperforated sinus membranes. The lack of difference in outcomes in these studies may be secondary to conscientious prevention and management of this common complication by oral surgeons, including repair of small defects with membrane folding, collagen tape, absorbable suturing, or fibrin glues.^15^ Several studies have advocated performing implant placement as a second stage procedure when membrane defects are >6 mm or when membrane defects >3 mm cannot be adequately closed during the primary surgery.^7,15-17^

For the otolaryngologist, perforation of the Schneiderian membrane is associated with an increased rate of sinus disease in patients. The literature indicates that 10% to 20% of patients with Schneiderian membrane perforation will experience transient or acute rhinosinusitis during their recovery from sinus lift surgery compared to 1% to 5% of patients with intact membranes.^6,7,9,12,13^ One review of 407 sinus lift surgeries noted a 10.5-fold (*P*<0.001) increase in the odds of developing postoperative sinusitis in patients who experienced a sinus membrane perforation intraoperatively.^11^ In the majority of patients, these infections were managed with oral antibiotics without the need for additional intervention. A study of 359 sinus membrane augmentations noted that the graft failure rate was substantially higher among patients requiring treatment for sinusitis postoperatively compared to patients without sinusitis: 30% vs 5%, respectively (*P*=0.007).^9^ These studies did not indicate that any patients required surgical intervention to treat their acute sinusitis.

A minority of patients undergoing a sinus lift procedure will progress to develop chronic rhinosinusitis. The estimated prevalence of chronic rhinosinusitis in sinus lift patients is estimated to be 4% to 8%.^4,5,13,18^ Limited publications in the otolaryngology literature examine this patient population, and study populations are generally small. Jiam et al examined 9 patients with chronic rhinosinusitis after a sinus lift procedure.^19^ Most patients presented within 3 months of surgery with nasal congestion, mucopurulent drainage, and facial pain that were nonresponsive to oral antibiotics. Four patients in the Jiam et al study cohort had sinus symptoms that persisted despite implant removal prior to otolaryngology evaluation, and 4 patients who did not undergo explantation prior to surgery had dental implants with direct continuity to the sinus cavity. All patients had resolution of sinus symptoms after surgery.^19^ These findings are consistent with the results of a study by Chen et al who reported complete resolution of symptoms in 15 patients requiring endoscopic sinus surgery for postoperative chronic rhinosinusitis.^20^ These findings are consistent with our study results. All 9 patients in this study developed ipsilateral post–sinus lift chronic rhinosinusitis that was refractory to medical management, required endoscopic sinus surgery, and had symptom resolution after surgery.

Eight of 9 patients in our patient population demonstrated potentially obstructive nasal anatomy that may have benefitted from surgical evaluation and potential correction before they underwent sinus lift surgery. Seven patients presented with septal deviation and 1 patient with a concha bullosa. Although only 3 patients required septoplasty and 1 patient required concha bullosectomy, our findings suggest that evaluation and possible treatment by an otolaryngologist may be beneficial in patients with obstructive anatomy on physical examination or imaging. None of the patients in this study had a pre–sinus lift CT scan of the sinuses, nasal endoscopy for assessment of sinonasal disease, or evaluation by an otolaryngologist.

Pignataro and colleagues have advocated a multidisciplinary approach to the management of candidates for the sinus lift procedure that includes preoperative risk stratification by the oral surgeon.^21^ This workup includes imaging of the sinuses; identification of risk factors (history of nasal trauma, preexisting symptoms of sinusitis, tobacco or illicit drug use, and immune-modifying processes); and medical and surgical optimization by an otolaryngologist as appropriate.^21^ Cote et al conducted a survey-based study examining otolaryngologists’ perspectives on preoperative assessment and found that a majority of otolaryngologists recommended a sinus CT scan prior to sinus lift.^22^ Otolaryngologists also recommended a referral to an ear, nose, and throat specialist for management of any findings that could contribute to perioperative sinus disease such as septal deviation with occlusion of the ostiomeatal complex, sinonasal mucosal thickening, sinus opacification, and evidence of oroantral fistula prior to proceeding to sinus lift surgery.^22^ Beaumont et al examined the prevalence of sinus disease and abnormalities among patients scheduled to undergo sinus augmentation and found that 27% of the patients had a history of sinus disease, and 40% of the patients were preoperatively diagnosed with sinonasal disease, including chronic rhinosinusitis, sinus cysts, nasal septum deviation, and ostium stenosis.^23^ However, the implications of preexisting sinus disease on imaging and physical examination are debatable. While Beaumont et al noted a high prevalence of preexisting sinonasal disease, they found that of their 45 patients, only 3 patients experienced postoperative complications, and 66% of complications occurred in patients without preexisting sinus disease. Ritter and colleagues noted similar findings in their patient population.^24^ They examined 145 patients undergoing sinus lift procedures and identified abnormalities on maxillary sinus imaging preoperatively in 46% of patients. They found that mucosal thickening was associated with lower rates of Schneiderian membrane perforation, and sinusitis was not observed in any patients regardless of presurgical imaging findings.^24^ Current studies do not provide clear guidance on the timing and the role of management of preexisting sinonasal disorders prior to sinus lift surgery.

The aim of this study was to identify and evaluate potential risk factors that should be considered prior to, during, and following sinus augmentation that may be predictive of acute or chronic sinusitis in patients. Sinusitis in the perioperative period is a somewhat common complication and not well-documented in the otolaryngology literature. The general otolaryngologist and the rhinologist must be aware of these risk factors as patients continue to undergo the sinus lift procedure and its variations.

One limitation of this study is that all patients were referred with a diagnosis of postoperative acute maxillary sinusitis. None of the patients was evaluated by an otolaryngologist prior to sinus lift surgery, so our ability to draw conclusions about the presence or absence of preoperative risk factors is limited; we examined patients after their complication had occurred. This study reflects one surgeon's experience and has the limitations inherent to small, retrospective, observational studies without a comparison group. Although 8 of 9 patients had evidence of possible contributory anatomic obstruction at the middle meatus at surgery, limited data were available regarding the patients’ histories prior to surgical intervention with sinus lift. Whether these patients had significant preexisting sinus disease or other risk factors that may have been identified preoperatively is unclear. The role and utility of preoperative otolaryngologic evaluation require further study. Future directions of research should include evaluating the time frame from sinus lift to sinus surgery, determining the recommended duration of medical treatment prior to surgical intervention, and performing a cost-benefit analysis of preoperative otolaryngology evaluation.

## CONCLUSION

Acute and chronic rhinosinusitis is a known complication of the sinus lift procedure and is more frequently seen in patients with a Schneiderian membrane perforation than in those with intact membranes. A minority of patients may eventually require surgical intervention by an otolaryngologist. Preoperative risk assessment may assist oral surgeons in identifying patients at risk for complications. Preoperative collaboration between oral surgeons and otolaryngologists may improve outcomes in patients undergoing sinus lift procedures.

## Notes

*This article meets the Accreditation Council for Graduate Medical Education and the American Board of Medical Specialties Maintenance of Certification competencies for Patient Care and Medical Knowledge.*
